# Poor health-related physical fitness performance increases the overweight and obesity risk in older adults from Taiwan

**DOI:** 10.1186/s12877-021-02112-1

**Published:** 2021-03-09

**Authors:** Chien-Chang Ho, Po-Fu Lee, Hui-Ling Chen, Ching-Yu Tseng, Xin-Yu Hsieh, Chih-Hui Chiu

**Affiliations:** 1 Department of Physical Education, Fu Jen Catholic University, New Taipei City, 242 Taiwan; 2 Research and Development Center for Physical Education, Health, and Information Technology, Fu Jen Catholic University, New Taipei City, 242 Taiwan; 3 Office of Physical Education Affairs, Taipei Medical University, Taipei City, 110 Taiwan; 4 Graduate Institute of Educational Leadership and Development, Fu Jen Catholic University, New Taipei City, 242 Taiwan; 5 Department of Exercise Health Science, National Taiwan University of Sport, Taichung City, Taiwan

**Keywords:** Physical fitness, Elders, Obesity, Body functioning

## Abstract

**Background:**

The purpose of the present study was to investigate the associations between health-related physical fitness performance and overweight/obesity risk among Taiwanese healthy older adults.

**Methods:**

A secondary dataset from the nationwide survey was applied in this study. Data from a total of 21,630 respondents aged 65–96 years were collected in this study. Demographic characteristics, life habits, perceived health status, anthropometric assessments, and health-related physical fitness measurements from this dataset were analyzed using the chi-square test, one-way analysis of variance, and logistic regression analysis.

**Results:**

The results indicated that overweight and obesity significantly associated with health-related physical fitness performance in the Taiwanese older adult population. In particular, the upper extremity muscular endurance scores of older adults with poor activity and physical fitness scores revealed obesity as a critical indicator of health-related physical fitness performance.

**Conclusions:**

Future studies can use muscle quality or body fat classification to predict obesity in older adults, which could more precisely portray the relationship between obesity and health-related physical fitness performance.

## Background

The increasing prevalence of obesity is a public concern in many developing and developed countries. Obesity increases the risk factors of various diseases, such as hypertension, heart diseases, cancer, and type II diabetes [1, 2]. Studies have indicated that obesity results in declining health-related physical fitness performance, including muscular endurance, muscular strength, flexibility, and cardiorespiratory capacity [3–5]. This decline reduces the quality of life and increases countries’ economic and social burden [6].

Aging has also been discovered to reduce health-related physical fitness performance. In the aging process, people’s muscular strength, muscle mass, cardiorespiratory fitness, and physical activity deteriorate while their body fat percentage increases, resulting in a risk of developing overweight and obesity in older adults [7, 8]. The older adults with BMI ≥ 30 kg/m^2^ had found to decrease muscle strength and function than normal weight older adults [9]. The reduction of physical tests was found in compromised with obesity older adults [10]. Furthermore, one study indicated that enhance one’s health-related physical fitness performance is significantly reducing the incidence of cardiovascular diseases [11]. In contrast, some studies have suggested that muscle mass plays the most important influence role in physical fitness performance rather than age or body fat [12]. Therefore, understanding the relationship between physical fitness and obesity in older adults is vital.

Although muscle strength and muscle endurance have been investigated a lot among older adults, the variations of their test performance on overweight/obesity risks have seldom examined. Besides, physical fitness including many aspects. It is warranted to investigate the relationship between physical fitness and overweight/obesity risks from different domains as well as its variations. Therefore, the present study aimed to determine the associations of health-related physical fitness performance with overweight and obesity risk among healthy older adults in Taiwan.

## Methods

### Study design and participants

The cross-sectional study was designed to analyze the physical fitness-based overweight/obesity risks. A secondary dataset from a nationwide survey (Taiwan’s National Physical Fitness Survey, TNPFS) was applied in the present study. Forty-six examination stations within 20 major cities or counties in Taiwan were responsible for the TNPFS data collection from 2014 to 2015. The TNPFS was supervised by the Sports Administration in Taiwan. The examiners of the TNPFS were qualified from the official training courses and capable for different types of physical fitness test (i.e., physical fitness test for adults aged 23 to 64, and for elder adults aged 65 years and above). All relevant data are contained as a secondary database and has been released for public research purposes. Previous studies have provided the detailed information of the TNPFS [13–15].

At first, 25,271 data from Taiwanese elders aged 65 years and above were included in 2014–15 TNPFS. However, in order to focus on the overweight/obese and normal weight (as the reference group) population, the data exclusion has applied according to their BMI classification. Finally, data from 21,630 healthy Taiwanese older adults aged 65 to 96 years were reviewed for the present study. This study’s design and analysis procedure was approved by The Institutional Review Board, Chung Shan Medical University Hospital (CS2–16114). All data used in this study was anonymous from the participants.

### Data collection

The TNPFS conducted the convenience sampling at each examination station. The data collection contained three different approaches. First, qualified examiners and medical specialists (usually nurse or doctor) were preliminary checked participants’ blood and resting heart rate, as well as assessed the potential safety risks by a structured questionnaire. All participants were required to pass the preliminary safety assessment then allowed to proceed next step. Second, participants were requested to fill (or verbally answer, if unavailable) the demographic questionnaire, as well as to complete the anthropometric measurements. After completed the second step, the participants were instructed for a light warming up (dynamic and static muscle stretching) around 10 min. Then, a series of physical fitness measurements were performed by the participants. Interval breaks are permitted for 2–4 min.

### Measurements

#### Demographic characteristics

The demographic questionnaire has previous described [13, 14]. It included personal characteristics (age, sex, education level, income, and marital), life habits (smoking and betel-nut chewing), and self-reported health status. This questionnaire was developed by Taiwan’s Sports Administration, Ministry of Education, and has implemented in the annual nationwide survey for years. The same questionnaire has also reported and published previously [13].

#### Anthropometric assessment

In this study, anthropometric assessments, including body weight (kg), height (m), waist, and hip circumference (WC & HC). All participants were required to remove their shoes and heavy clothes during measuring. Body mass index (BMI) of the participants then calculated (kg/m^2^). The WC and HC were measured to the nearest 0.1 cm by a soft measuring tape at the natural waist and greater trochanter level. Thus, it allowed the waist-to-hip ratio (WHR) to be calculated. The cut-off values for BMI were suggested by the Taiwanese Ministry of Health and Welfare [16]. However, as mentioned above, only normal weight (18.5 to 23.99 kg/m^2^), overweight (24 to 26.99 kg/m^2^), and obesity (27 kg/m^2^ and above) population were included for present study.

#### Physical fitness measurements

The step test (reps in 2 min), arm curl (reps in 30 s), chair stand (reps in 30 s), back scratch (cm), chair sit-and-reach (cm), one-leg stance with eye open (seconds), and 8-ft up-and-go (seconds) were measured to access the functional capacity among the participants. These measurements were representing participants’ cardio-endurance, muscle strength and endurance, body flexibility, and balance ability. The protocol of these measurements was conducted by the qualified examiners. Besides, the protocol of these measurements was mainly performed by the Senior Fitness Test manual [17], except the one-leg stance with eye open was performed according to the previous approach [18]. The results of these measurements were classified respectively into four quartiles for further analysis.

### Statistical analyses

The SAS software (Statistical Analysis System; version 9.4, SAS Institute Inc., Cary, NC) was applied in this study. Demographic characteristics and physical fitness measurements were analyzed for Chi-square tests, one-way analysis of variance (ANOVA), and Tukey’s post hoc tests among the groups. Significant differences between groups are considered as the potential confounders for the logistic regression model adjustment. Further, the normal weight population was appointed as the reference group for logistic regression analyses for general physical fitness test results as well as the quartiles of the results. Thus, the overweight and obesity risks (ORs) of each physical fitness performance were estimated, respectively. All values were expressed as means ± standard deviation, or the percentage (frequency). The significant level within each analysis was *p* <  0.05 to reject the null hypothesis and with a confidence interval (CI) of 95%.

## Results

Twenty-one thousand six hundred thirty participants aged 65 to 96 years with complete data were included from Taiwan’s National Physical Fitness Examination Survey Databases (35.38% of men). Demographic characteristics and anthropometric variables are presented in Table 1. The highest proportion of general obesity status was normal weight (41.69%). All participants were divided into trichotomy groups as normal weight, overweight, and obesity by gender. The significant differences were shown between normal weight, overweight, and obesity groups on all relevant variables except men’s marital status (*p* = 0.481). In contrast, there were significant differences between normal weight, overweight, and obesity groups on all relevant variables except smoking status in women (*p* = 0.236).

**Table 1:** Characteristics of the study participants according to general obesity status in Taiwanese older adults

Variables	Men (*N* = 7652)				Women (*N* = 13,978)			
	Obesity (*n* = 1671)	Overweight (*n* = 2840)	Normal Weight (*n* = 3141)	*p*	Obesity (*n* = 3534)	Overweight (*n* = 4568)	Normal weight (*n* = 5876)	*p*
Age (%)				< 0.001*				< 0.001*
65–69 years old	32.50	30.56	27.54		36.64	37.06	37.25	
70–74 years old	25.79	25.35	24.90		29.20	28.63	26.92	
75–79 years old	23.16	20.53	21.59		21.62	20.56	18.98	
80–84 years old	11.13	14.40	14.93		9.42	9.96	11.15	
≧ 85 years old	7.42	9.15	11.05		3.11	3.79	5.70	
Height (cm)	162.17 ± 5.79	163.47 ± 5.83	163.74 ± 6.11	< 0.001*	151.63 ± 5.60	152.69 ± 5.66	153.21 ± 5.80	< 0.001*
Body weight (kg)	75.67 ± 5.77	67.95 ± 5.31	59.25 ± 5.78	< 0.001*	67.74 ± 6.36	59.27 ± 4.79	51.54 ± 4.99	< 0.001*
BMI (kg/m^2^)	28.76 ± 1.43	25.40 ± 0.87	22.07 ± 1.38	< 0.001*	29.44 ± 2.04	25.39 ± 0.86	21.93 ± 1.40	< 0.001*
WC (cm)	97.50 ± 6.44	90.69 ± 5.83	83.14 ± 6.33	< 0.001*	93.40 ± 7.52	85.99 ± 7.00	79.06 ± 7.08	< 0.001*
HC (cm)	101.69 ± 4.55	97.30 ± 4.46	92.62 ± 4.51	< 0.001*	102.63 ± 5.24	97.21 ± 4.77	91.97 ± 4.58	< 0.001*
WHR	0.96 ± 0.06	0.93 ± 0.05	0.90 ± 0.06	< 0.001*	0.91 ± 0.07	0.89 ± 0.07	0.86 ± 0.07	< 0.001*
Education level (%)				< 0.001*				< 0.001*
Elementary school or lower	50.89	42.28	40.96		72.92	66.67	57.74	
Junior or senior school	30.19	34.83	32.54		21.99	25.57	30.18	
College or higher	18.92	22.89	26.50		5.09	7.76	12.07	
Income level (%)				0.025*				< 0.001*
≦ 20,000 NTD	81.86	78.78	78.11		93.35	91.59	87.86	
20,001–40,000 NTD	10.04	11.94	11.23		4.52	5.30	7.61	
≧ 40,001 NTD	8.10	9.28	10.66		2.12	3.11	4.54	
Marital status (%)				0.481				0.001*
Never married	58.49	59.67	58.08		48.40	47.18	50.85	
Married	30.69	30.06	30.22		24.84	27.05	24.41	
Divorced/separation/widowed	10.82	10.28	11.70		26.75	25.77	24.75	
Self-reported health status (%)				0.038*				< 0.001*
Excellent or good	65.44	69.44	69.14		61.93	65.08	64.22	
Fair	26.55	24.28	24.32		28.16	26.53	28.20	
Very bad or poor	8.02	6.27	6.54		9.91	8.39	7.59	
Smoking status (%)				0.012*				0.236
Never	78.74	82.20	80.27		97.33	97.62	97.27	
Current	11.51	9.84	11.92		1.60	1.71	1.95	
Former	9.76	7.97	7.80		1.07	0.66	0.79	
Chewing betel nut (%)				< 0.001*				< 0.001*
Never	93.17	95.03	96.11		98.33	99.11	99.12	
Current	2.63	1.84	1.30		1.19	0.66	0.47	
Former	4.20	3.13	2.60		0.48	0.23	0.41	

Values are expressed as means ± standard deviation

*Abbreviations: BMI* body mass index, *HC* hip circumference, *NTD* New Taiwan Dolloar, *WC* waist circumference, *WHR* waist-to-hip ratio

^*^p <  0.05

Table 2 presented the comparison of inter-group differences by various health-related physical fitness measurements. All the general obesity status groups were significant differences in all the health-related physical fitness measurements in both men and women, and obesity individuals got the lowest grade of all measurements except the 30-s arm curl test.

**Table 2:** Health-related physical fitness measurements according to general obesity status in Taiwanese older adults

Variables	Obesity	Overweight	Normal weight	*p*	Tukey’s post hoc test
**Men**					
2-min step test (step)	85.33 ± 23.70	88.33 ± 23.01	87.68 ± 23.98	< 0.001*	Obesity < Overweight, Normal weight
30-s arm curl test (rep)	17.92 ± 5.44	17.90 ± 5.68	17.37 ± 5.69	< 0.001*	Obesity, Overweight > Normal weight
30-s chair stand test (rep)	14.67 ± 4.58	15.33 ± 4.94	15.35 ± 4.92	< 0.001*	Obesity < Overweight, Normal weight
Back scratch test (cm)	−15.14 ± 12.73	−11.90 ± 12.54	−8.47 ± 12.31	< 0.001*	Obesity < Overweight < Normal weight
Chair sit-and-reach test (cm)	2.12 ± 8.17	2.69 ± 8.45	2.80 ± 8.32	0.035*	Obesity < Normal weight
8-ft up-and-go test (s)	7.46 ± 1.88	7.11 ± 1.86	7.04 ± 1.88	< 0.001*	Obesity > Overweight, Normal weight
One-leg stance with eye open test (s)	14.11 ± 11.17	15.93 ± 11.64	16.62 ± 11.63	< 0.001*	Obesity < Overweight, Normal weight
**Women**					
2-min step test (step)	81.97 ± 24.43	84.77 ± 23.82	86.50 ± 23.84	< 0.001*	Obesity < Overweight < Normal weight
30-s arm curl test (rep)	17.33 ± 5.47	17.50 ± 5.33	17.05 ± 5.61	< 0.001*	Obesity, Overweight > Normal weight
30-s chair stand test (rep)	13.83 ± 4.42	14.65 ± 4.49	15.05 ± 4.78	< 0.001*	Obesity < Overweight < Normal weight
Back scratch test (cm)	−8.79 ± 11.28	−5.26 ± 10.48	−2.49 ± 10.12	< 0.001*	Obesity < Overweight < Normal weight
Chair sit-and-reach test (cm)	4.64 ± 7.73	5.56 ± 7.90	5.82 ± 8.10	< 0.001*	Obesity < Overweight, Normal weight
8-ft up-and-go test (s)	7.89 ± 1.86	7.38 ± 1.74	7.18 ± 1.82	< 0.001*	Obesity > Overweight > Normal weight
One-leg stance with eye open test (s)	11.84 ± 10.40	14.21 ± 10.91	16.01 ± 11.45	< 0.001*	Obesity < Overweight < Normal weight

Values are expressed as means ± standard deviation

*p < 0.05

Table 3 represented the multivariate adjusted ORs for overweight in relation to health-related physical fitness measurements after adjustment for potential confounders. Statistical significant was found on the back scratch test in both men and women groups before adjustment (OR = 0.98, 95% CI: 0.97–0.98; OR = 0.97, 95% CI: 0.97–0.98). The significant of 30-s arm test, 30-s chair stand test and one-leg stance with eye open test was founded in women particularly (OR = 1.03, 95% CI: 1.02–1.04; OR = 0.98, 95% CI: 0.97–0.99; OR = 0.99, 95% CI: 0.99–0.99). After confounders adjusted back scratch test were still significantly in both men and women (OR = 0.98, 95% CI: 0.97–0.99; OR = 0.98, 95% CI: 0.98–0.99), and 30-s arm curl test were significant too in women (OR = 1.02, 95% CI: 1.01–1.03). Particularly, Chair sit-and-reach test become significantly in both men and women (OR = 1.02, 95% CI: 1.01–1.03; OR = 1.01, 95% CI: 1.01–1.02). Table 4 represented the multivariate adjusted ORs for obesity in relation to health-related physical fitness measurements after adjustment for potential confounders. Statistical significant was found on the 30-s arm curl test and back scratch test in both men and women groups before adjustment (men OR = 1.04, 95% CI: 1.03–1.06; OR = 0.96, 95% CI: 0.95–0.96; women OR = 1.04, 95% CI: 1.03–1.05; OR = 0.95, 95% CI: 0.94–0.95). The significant of 30-s chair stand test, 8-ft up-and-go test and one-leg stance with eye open test was founded in women particularly (OR = 0.97, 95% CI: 0.96–0.99; OR = 1.08, 95% CI: 1.05–1.12; OR = 0.98, 95% CI: 0.98–0.99). After confounders adjusted 30-s arm curl test and back scratch test were still significantly (men OR = 1.04, 95% CI: 1.01–1.06; OR = 0.97, 95% CI 0.96–0.98; women OR = 1.03, 95% CI: 1.01–1.05; OR = 0.97, 95% CI: 0.96–0.97), and ORs of the chair sit-and-reach test increased and became significant in both men and women (OR = 1.04, 95% CI: 1.03–1.06; OR = 1.01, 95% CI: 1.00–1.02). Additional, ORs of the one-leg stance with eye open test increased and still significant after adjusting in women (OR = 0.99, 95% CI: 0.98–1.00).

**Table 3:** Multivariate adjusted odds ratios (OR) and 95% confidence intervals (95% CI) for overweight in relation to health-related physical fitness measurements after adjustment for potential confounders

Variables	Model 1 (unadjusted)			Model 2 (adjusted^**a**^)		
	OR	95% CI	*p*	OR	95% CI	*p*
**Men**						
2-min step test (step)	1.00	0.99–1.00	0.358	1.00	0.99–1.00	0.802
30-s arm curl test (rep)	1.02	1.01–1.03	0.002	1.01	0.99–1.02	0.449
30-s chair stand test (rep)	0.99	0.98–1.01	0.412	1.01	0.99–1.03	0.515
Back scratch test (cm)	0.98	0.97–0.98	< 0.001*	0.98	0.97–0.99	< 0.001*
Chair sit-and-reach test (cm)	1.00	0.99–1.01	0.235	1.02	1.01–1.03	0.001*
8-ft up-and-go test (s)	0.99	0.95–1.03	0.594	0.98	0.92–1.03	0.392
One-leg stance with eye open test (s)	1.00	0.99–1.00	0.392	1.01	0.99–1.02	0.070
**Women**						
2-min step test (step)	1.00	0.99–1.00	0.121	1.00	0.99–1.00	0.586
30-s arm curl test (rep)	1.03	1.02–1.04	< 0.001*	1.02	1.01–1.03	< 0.001*
30-s chair stand test (rep)	0.98	0.97–0.99	0.011*	1.00	0.98–1.01	0.714
Back scratch test (cm)	0.97	0.97–0.98	< 0.001*	0.98	0.98–0.99	< 0.001*
Chair sit-and-reach test (cm)	1.01	0.99–1.01	0.104	1.01	1.01–1.02	< 0.001*
8-ft up-and-go test (s)	1.00	0.97–1.03	0.916	0.97	0.93–1.01	0.113
One-leg stance with eye open test (s)	0.99	0.99–0.99	< 0.001*	1.00	0.99–1.00	0.223

^a^Adjusted for age, waist circumference, education, monthly income, marital status, self-reported health status, smoking status, and chewing betel nuts

*p < 0.05

**Table 4:** Multivariate adjusted odds ratios (OR) and 95% confidence intervals (95% CI) for obese in relation to health-related physical fitness measurements after adjustment for potential confounders

Variables	Model 1 (unadjusted)			Model 2 (adjusted^**a**^)		
	OR	95% CI	*p*	OR	95% CI	*p*
**Men**						
2-min step test (step)	1.00	0.99–1.00	0.928	1.00	0.99–1.01	0.443
30-s arm curl test (rep)	1.04	1.03–1.06	< 0.001*	1.04	1.01–1.06	0.009*
30-s chair stand test (rep)	0.99	0.97–1.01	0.163	1.01	0.98–1.05	0.536
Back scratch test (cm)	0.96	0.95–0.96	< 0.001*	0.97	0.96–0.98	< 0.001*
Chair sit-and-reach test (cm)	1.00	0.99–1.01	0.363	1.04	1.03–1.06	< 0.001*
8-ft up-and-go test (s)	1.05	0.99–1.10	0.054	1.00	0.92–1.09	0.976
One-leg stance with eye open test (s)	0.99	0.99–1.00	0.069	1.01	0.99–1.02	0.359
**Women**						
2-min step test (step)	1.00	0.99–1.00	0.818	1.00	0.99–1.01	0.445
30-s arm curl test (rep)	1.04	1.03–1.05	< 0.001*	1.03	1.01–1.05	< 0.001*
30-s chair stand test (rep)	0.97	0.96–0.99	< 0.001*	1.00	0.98–1.02	0.895
Back scratch test (cm)	0.95	0.94–0.95	< 0.001*	0.97	0.96–0.97	< 0.001*
Chair sit-and-reach test (cm)	1.00	0.99–1.01	0.453	1.01	1.00–1.02	0.014*
8-ft up-and-go test (s)	1.08	1.05–1.12	< 0.001*	1.05	0.99–1.11	0.108
One-leg stance with eye open test (s)	0.98	0.98–0.99	< 0.001*	0.99	0.98–1.00	0.008*

^a^Adjusted for age, waist circumference., education, monthly income, marital status, self-reported health status, smoking status, and chewing betel nuts

*p < 0.05

According to this, Table 5 presented the results of the logistic regression analyses. The quartile with the best performance was appointed to be a reference for all regression for analysis (OR = 1.00), except the 8-ft up-and-go test appointed the lowest performance as a reference. After factor adjusted by potential confounders, statistic evidence pointed that men participants who performed in the first level of the 2-min step test (< 73 steps) and 30-s arm curl test (< 14 rep) were associated with the risk of the overweight (OR = 0.79, 95% CI: 0.64–0.98; OR = 0.80, 95% CI: 0.64–0.99) compared with the reference group, whereas all the level of chair sit-and-reach test were associated with the risk of the overweight and third level had the highest risk of the overweight (OR = 0.81, 95% CI: 0.67–0.99), and all the level of back scratch test were associated with the risk of the overweight and first level (< − 21) had the highest risk of the overweight (OR = 2.55, 95% CI: 2.04–3.18) compared with the reference group. In females, participants with the lowest performance on the 30-s arm curl test had the highest risk of the overweight (OR = 0.81, 95% CI: 0.90–0.95). Specifically, females in the best performance group on chair sit-and-reach test (4–11) had the highest risk of the overweight (OR = 0.86, 95% CI: 0.75–0.99). Performed in the second level of the back scratch test (− 12 ~ − 2) and one-leg stance with eye open test (4.4–10.7) had the highest risk of the overweight compared with the reference group (OR = 2.04, 95% CI: 1.75–2.37; OR = 1.18, 95% CI: 1.02–1.37).

**Table 5:** Multivariate adjusted odds ratios (OR) and 95% confidence intervals (95% CI) for overweight in relation to quartiles of health-related physical fitness measurements after adjustment for potential confounders

Variables	Model 1 (unadjusted)			Model 2 (adjusted^**a**^)		
	OR	95% CI	*p*	OR	95% CI	*p*
**Men**						
2-min step test (step)						
< 73	0.84	0.71–0.99	0.037*	0.79	0.64–0.98	0.031*
73–89	1.00	0.85–1.17	0.962	0.87	0.71–1.08	0.204
90–103	0.99	0.85–1.15	0.840	0.89	0.74–1.08	0.255
> 103	1.00	–	–	1.00	–	–
Test for trend	0.037*			0.029*		
30-s arm curl test (rep)						
< 14	0.72	0.61–0.85	< 0.001*	0.80	0.64–0.99	0.039*
14–17	0.85	0.72–1.00	0.0497*	0.87	0.70–1.08	0.198
18–22	0.89	0.76–1.04	0.148	0.98	0.81–1.20	0.873
> 22	1.00	–	–	1.00	–	–
Test for trend	< 0.001*			0.021*		
30-s chair stand test (rep)						
< 12	1.02	0.85–1.22	0.826	0.94	0.75–1.19	0.599
12–14	1.02	0.86–1.20	0.854	1.04	0.83–1.30	0.731
15–18	1.04	0.89–1.21	0.645	0.97	0.80–1.18	0.754
> 18	1.00	–	–	1.00	–	–
Test for trend	0.985			0.652		
Back scratch test (cm)						
< −21	2.84	2.40–3.36	< 0.001*	2.55	2.04–3.18	< 0.001*
− 21 – −11	2.01	1.72–2.35	< 0.001*	1.73	1.41–2.12	< 0.001*
-10–0	1.86	1.60–2.15	< 0.001*	1.81	1.50–2.19	< 0.001*
> 0	1.00	–	–	1.00	–	–
Test for trend	< 0.001*			< 0.001*		
Chair sit-and-reach test (cm)						
< −2	0.86	0.74–1.00	0.057	0.63	0.52–0.78	< 0.001*
-2–0	0.81	0.68–0.96	0.015*	0.64	0.51–0.79	< 0.001*
1–8	0.90	0.77–1.04	0.161	0.81	0.67–0.99	0.036*
> 8	1.00	–	–	1.00	–	–
Test for trend	0.050			< 0.001*		
8-ft up-and-go test (s)						
< 5.8	1.01	0.86–1.19	0.923	1.07	0.86–1.33	0.530
5.8–6.8	1.11	0.94–1.32	0.228	1.12	0.90–1.41	0.307
6.9–8.3	1.09	0.93–1.28	0.303	1.08	0.88–1.34	0.456
> 8.3	1.00	–	–	1.00	–	–
Test for trend	0.624			0.767		
One-leg stance with eye open test (s)						
< 5.0	1.02	0.71–1.46	0.923	0.64	0.39–1.04	0.069
5.0–12.7	1.04	0.73–1.49	0.836	0.74	0.46–1.19	0.219
12.8–30.0	0.90	0.63–1.27	0.537	0.68	0.43–1.07	0.094
> 30.0	1.00	–	–	1.00	–	–
Test for trend	0.159			0.314		
**Women**						
2-min step test (step)						
< 69	1.06	0.94–1.21	0.356	0.97	0.83–1.13	0.668
69–87	1.10	0.98–1.25	0.107	1.01	0.88–1.18	0.850
88–101	1.09	0.97–1.22	0.168	1.04	0.90–1.20	0.598
> 101	1.00	–	–	1.00	–	–
Test for trend	0.604			0.469		
30-s arm curl test (rep)						
< 14	0.73	0.64–0.82	< 0.001*	0.81	0.70–0.95	0.008*
14–16	0.84	0.74–0.96	0.012*	0.98	0.83–1.15	0.761
17–21	0.93	0.83–1.04	0.211	0.99	0.86–1.14	0.838
> 21	1.00	–	–	1.00	–	–
Test for trend	< 0.001*			0.002*		
30-s chair stand test (rep)						
< 11	1.10	0.95–1.28	0.219	0.96	0.80–1.16	0.693
11–13	1.16	1.01–1.33	0.032*	1.00	0.85–1.19	0.974
14–18	1.16	1.03–1.30	0.013*	1.06	0.92–1.23	0.393
> 18	1.00	–	–	1.00	–	–
Test for trend	0.578			0.310		
Back scratch test (cm)						
< −12	2.50	2.19–2.86	< 0.001*	1.73	1.46–2.04	< 0.001*
-12 – −2	2.39	2.11–2.70	< 0.001*	2.04	1.75–2.37	< 0.001*
-1–3	1.60	1.43–1.80	< 0.001*	1.44	1.25–1.66	< 0.001*
> 3	1.00	–	–	1.00	–	–
Test for trend	< 0.001*			< 0.001*		
Chair sit-and-reach test (cm)						
< 0	0.81	0.72–0.92	0.002*	0.69	0.59–0.81	< 0.001*
0–3	0.97	0.87–1.09	0.619	0.87	0.76–1.00	0.054
4–11	0.95	0.85–1.07	0.425	0.86	0.75–0.99	0.036*
> 11	1.00	–	–	1.00	–	–
Test for trend	0.003*			< 0.001*		
8-ft up-and-go test (s)						
< 6.0	0.89	0.78–1.01	< 0.001*	0.93	0.79–1.09	0.376
6.0–7.0	1.08	0.95–1.23	0.064	1.13	0.96–1.32	0.137
7.1–8.6	1.08	0.95–1.22	0.230	1.05	0.90–1.22	0.578
> 8.6	1.00	–	–	1.00	–	–
Test for trend	0.002*			0.168		
One-leg stance with eye open test (s)						
< 4.4	1.20	1.06–1.36	0.005*	0.99	0.84–1.16	0.868
4.4–10.7	1.34	1.19–1.50	< 0.001*	1.18	1.02–1.37	0.028*
10.8–27.0	1.24	1.10–1.38	< 0.001*	1.10	0.96–1.26	0.188
> 27.0	1.00	–	–	1.00	–	–
Test for trend	0.004*			0.933		

^a^Adjusted for age, waist circumference, education, monthly income, marital status, self-reported health status, smoking status, and chewing betel nuts

*p < 0.05

The results of the logistic regression models for the risk of obesity were shown in Table 6. After factor adjusted by potential confounders, the result showed that participants who performed in the first level of the 30-s arm curl test (< 14 rep) and back scratch test (< − 21 cm) had the highest risk of the obesity (OR = 0.65, 95% CI: 0.45–0.93; OR = 3.49, 95% CI: 2.40–5.09) compared with the reference group, whereas all the level of chair sit-and-reach test were associated with the risk of the obesity and third level had the highest risk of the obesity (OR = 0.65, 95% CI: 0.47–0.91) in men. Performed in the second level on 30-s chair stand test (12–14 rep) and one-leg stance with eye open test (5.0–12.7 s) were associated with the risk of the obesity and had the higher risk of the obesity (OR = 1.46, 95% CI: 1.01–2.11; OR = 0.42, 95% CI: 0.20–0.89) than the reference group in men. In the case of females, participants with the lowest performance on 30-s arm curl test (< 14) and 30-s chair stand test (< 11) had the highest risk of the obesity (OR = 0.79, 95% CI: 0.63–0.98; OR = 0.72, 95% CI: 0.55–0.94) compared with the reference group. Conversely, the best level of the 8-ft up-and-go test (< 6) had the highest risk of the obesity (OR = 0.66, 95% CI: 0.53–0.82). Performed in the second level on chair sit-and-reach test (0–3), back scratch test (− 12- -2) and one-leg stance with eye open test (4.4–10.7) had the higher risk of the obesity (OR = 0.81, 95% CI: 0.66–1.00; OR = 3.47, 95% CI: 2.75–4.38; OR = 1.26, 95% CI: 1.01–1.56) compared with the reference group.

**Table 6:** Multivariate adjusted odds ratios (OR) and 95% confidence intervals (95% CI) for obese in relation to quartiles of health-related physical fitness measurements after adjustment for potential confounders

Variables	Model 1 (unadjusted)			Model 2 (adjusted^**a**^)		
	OR	95% CI	*p*	OR	95% CI	*p*
**Men**						
2-min step test (step)						
< 73	1.02	0.84–1.25	0.814	0.69	0.48–1.00	0.051
73–89	1.16	0.95–1.41	0.146	0.92	0.65–1.31	0.651
90–103	1.05	0.86–1.27	0.649	0.91	0.66–1.27	0.578
> 103	1.00	–	–	1.00	–	–
Test for trend	0.705			0.096		
30-s arm curl test (rep)						
< 14	0.62	0.51–0.77	< 0.001*	0.65	0.45–0.93	0.020*
14–17	0.74	0.61–0.91	0.004*	0.64	0.44–0.92	0.015*
18–22	0.90	0.75–1.09	0.287	0.87	0.62–1.22	0.423
> 22	1.00	–	–	1.00	–	–
Test for trend	< 0.001*			0.007*		
30-s chair stand test (rep)						
< 12	1.07	0.86–1.33	0.535	0.94	0.64–1.39	0.765
12–14	1.16	0.94–1.42	0.170	1.46	1.01–2.11	0.045*
15–18	1.08	0.89–1.30	0.454	1.20	0.86–1.67	0.297
> 18	1.00	–	–	1.00	–	–
Test for trend	0.648			0.833		
Back scratch test (cm)						
< −21	5.24	4.24–6.49	< 0.001*	3.49	2.40–5.09	< 0.001*
-21 – −11	3.22	2.62–3.96	< 0.001*	2.21	1.54–3.16	< 0.001*
-10–0	2.22	1.81–2.72	< 0.001*	1.80	1.27–2.54	0.001*
> 0	1.00	–	–	1.00	–	–
Test for trend	< 0.001*			< 0.001*		
Chair sit-and-reach test (cm)						
< −2	0.87	0.72–1.04	0.130	0.34	0.24–0.49	< 0.001*
-2–0	0.90	0.74–1.11	0.316	0.49	0.34–0.71	< 0.001*
1–8	0.90	0.74–1.11	0.250	0.65	0.47–0.91	0.012*
> 8	1.00	–	–	1.00	–	–
Test for trend	0.195			< 0.001*		
8-ft up-and-go test (s)						
< 5.8	0.81	0.67–0.98	0.028	1.07	0.75–1.53	0.701
5.8–6.8	1.03	0.84–1.25	0.788	1.01	0.70–1.46	0.967
6.9–8.3	1.10	0.92–1.33	0.290	1.07	0.77–1.50	0.686
> 8.3	1.00	–	–	1.00	–	–
Test for trend	0.002*			0.838		
One-leg stance with eye open test (s)						
< 5.0	0.86	0.57–1.31	0.491	0.32	0.15–0.69	0.003*
5.0–12.7	0.88	0.58–1.33	0.543	0.42	0.20–0.89	0.023*
12.8–30.0	0.66	0.44–0.99	0.047*	0.34	0.16–0.70	0.004*
> 30.0	1.00	–	–	1.00	–	–
Test for trend	0.033*			0.197		
**Women**						
2-min step test (step)						
< 69	1.08	0.94–1.24	0.273	0.93	0.75–1.16	0.517
69–87	1.01	0.89–1.16	0.837	0.87	0.70–1.07	0.188
88–101	0.87	0.76–1.00	0.043*	0.87	0.71–1.07	0.198
> 101	1.00	–	–	1.00	–	–
Test for trend	0.131			0.575		
30-s arm curl test (rep)						
< 14	0.64	0.55–0.73	< 0.001*	0.79	0.63–0.98	0.030*
14–16	0.67	0.58–0.78	< 0.001*	0.88	0.69–1.11	0.276
17–21	0.81	0.94–1.24	0.001*	0.87	0.71–1.06	0.161
> 21	1.00	–	–	1.00	–	–
Test for trend	< 0.001*			0.022*		
30-s chair stand test (rep)						
< 11	1.16	0.98–1.38	0.091	0.72	0.55–0.94	0.017*
11–13	1.27	1.08–1.48	0.003*	0.91	0.71–1.16	0.425
14–18	1.22	1.06–1.40	0.005*	0.92	0.75–1.14	0.466
> 18	1.00	–	–	1.00	–	–
Test for trend	0.395			0.009*		
Back scratch test (cm)						
< −12	5.32	4.55–6.21	< 0.001*	3.10	2.43–3.94	< 0.001*
-12 – −2	4.13	3.56–4.80	< 0.001*	3.47	2.75–4.38	< 0.001*
-1–3	1.84	1.59–2.14	< 0.001*	1.64	1.31–2.07	< 0.001*
> 3	1.00	–	–	1.00	–	–
Test for trend	< 0.001*			< 0.001*		
Chair sit-and-reach test (cm)						
< 0	0.83	0.72–0.96	0.013*	0.64	0.51–0.80	< 0.001*
0–3	0.95	0.83–1.08	0.428	0.81	0.66–1.00	0.049*
4–11	0.96	0.84–1.10	0.543	0.94	0.76–1.16	0.556
> 11	1.00	–	–	1.00	–	–
Test for trend	0.017*			< 0.001*		
8-ft up-and-go test (s)						
< 6.0	0.60	0.53–0.69	< 0.001*	0.66	0.53–0.82	< 0.001*
6.0–7.0	0.82	0.72–0.94	0.006*	0.86	0.69–1.08	0.193
7.1–8.6	0.94	0.82–1.07	0.352	0.91	0.74–1.12	0.374
> 8.6	1.00	–	–	1.00	–	–
Test for trend	< 0.001*			< 0.001*		
One-leg stance with eye open test (s)						
< 4.4	1.78	1.54–2.05	< 0.001*	1.17	0.93–1.48	0.180
4.4–10.7	1.56	1.36–1.79	< 0.001*	1.26	1.01–1.56	0.038*
10.8–27.0	1.39	1.22–1.59	< 0.001*	1.17	0.95–1.44	0.153
> 27.0	1.00	–	–	1.00	–	–
Test for trend	< 0.001*			0.206		

^a^Adjusted for age, waist circumference, education, monthly income, marital status, self-reported health status, smoking status, and chewing betel nuts

*p < 0.05

## Discussion

This study employed large-sample national survey data to discuss the relationship between functional physical fitness and obesity risks in older adults. The results indicated that overweight and obesity significantly reduced the health-related physical fitness performance in a Taiwanese older adult population. In particular, the upper extremity muscular endurance scores of older adults with poor activity and physical fitness scores revealed obesity to be a critical indicator of health-related physical fitness performance. These findings are critical for establishing health care policies in the future.

Obesity is a key influential factor in the flexibility in older adults in Taiwan. overweight and obesity are critical indicators of higher percentages of body fat and visceral fat, resulting in aggravated low-grade inflammation and osteoarthritis, further affecting the flexibility in older adults [19, 20]. Studies have verified that aging decreases the flexibility [21]. In particular, the BMI and flexibility in female older adults exhibit a significant negative correlation, which indicates that overweight and obesity reduce the flexibility [22–24]. This study used interference factors to adjust and compute odds ratios; the findings revealed that the lower flexibility scores are associated with higher overweight or obese risks. Specifically, the odds ratios for this relationship, especially for back scratch test, were listed from 1.44 to 3.49. This indicated a 44 to 249% of exceeded odds for overweight/obese could be performed by the low body flexibility population when compare with reference group. The back scratch test could be used as a preliminary check for elders’ overweight/obese risk.

Besides, this study discovered that obesity had different influences on upper extremity muscular endurance and lower extremity muscle strength and endurance in older adults. This study adjusted the research data for age, WC, education, monthly income, marital status, self-reported health status, smoking status, and betel nut chewing status for the influences on lower extremity muscle strength and endurance. Our findings revealed that older adults with normal weight had more favorable lower extremity muscular strength (8 Foot Up-and-Go test) and muscular endurance (30-s chair stand test) scores. However, the scores for overweight and obesity older adults did not exhibit significant differences. This result is consistent with that of the previous study [25]. In this study, Brady (2014) measured participants’ leg press strength and divided the scores by their lower extremities’ weight to quantify their muscle quality. The results revealed that healthy older adults’ muscle quality was significantly higher than that of overweight and obesity older adults, with no significant differences in muscle quality observed between the latter two [25]. This result is consistent with that of the present study, namely that the 8 ft up-and-go and 30-s chair stand test results of overweight and obesity older adults did not exhibit significant differences. Therefore, BMI is a viable indicator for predicting older adults’ obesity level, but cannot serve as an indicator for predicting their lower extremity muscular strength and endurance. A possible reason for this result may be older adults’ different body densities because BMI values cannot wholly reflect the proportions of an individual’s muscles and fat [26].

Unlike the results on lower extremities, this study revealed that obesity significantly influenced older adults’ upper extremity muscular endurance. This was particularly true for overweight female older adults and the obesity population. After conducting logistic regression and making adjustments for potential confounders, the present researchers discovered that overweight and obesity posed higher risks to the first and second levels in this study; by contrast, the other two intervals did not exhibit significant differences. These results indicated that obesity and overweight risk is significantly lower for older adults with more favorable upper extremity muscular endurance. Therefore, physical activity interventions can enhance exercise capabilities, significantly reducing the adverse effects of obesity on upper extremity muscular endurance.

The results revealed that for older adults in Taiwan, the 2-min step test results of overweight and obesity older adults are significantly worse than those of older adults of normal weight. However, after being adjusted by interference factors, this phenomenon was only true for overweight male patients who had stepped on a ladder < 73 times. This result indicated that weight influences male older adult populations with poor aerobic physical fitness, but not women or obesity populations. This finding is inconsistent with studies that have discovered the influence of obesity on aerobic exercise capabilities [5, 11]. However, this inconsistency could be caused by differences in participants’ age, sex, and ethnicity, interference factors, or statistical power value. Future studies can discuss the relationship between other aerobic physical fitness indicators and overweight and obesity.

On the other hand, the use of BMI cutoffs was based on a Taiwanese normative value. Although it was suggested by the Ministry of Health and Welfare in Taiwan, the cutoffs decided the grouping of the research population. This may be a critical factor to determine the results. The international cutoffs are suggested for future studies to examine the association between elders’ physical fitness test performance and overweight/obese risks.

The strength of the present study was using a representative database. Although the potential confounders were considered throughout the analysis, some limitations should be addressed. First, the data used in this study was mainly included Chinese Taiwanese population. Future studies should investigate the populations from different races, lifestyles and cultures, social-economic backgrounds, etc. Second, the use of a secondary database limited the possibility of discussing other elder-related factors, such as chronic diseases, dietary and nutrition, and living status. These factors critically influence the elder’s quality of life as well as their health status. Furthermore, the daily physical activities participation was unable to discuss, either. Future studies are suggested to proceed with these aspects. Third, due to cross-sectional study design was applied, there is no cause and effect relationship can be guaranteed. Future studies may conduct longitudinal studies to have a better understanding of this relationship.

## Conclusion

In conclusion, this study discovered the relationship between Taiwanese elders’ physical fitness and their overweight/obese risks. Furthermore, the findings revealed that BMI to be a classification standard for overweight and obesity. However, it was incapable of precisely predicting the effects of obesity on lower extremity muscular strength, muscular endurance, and aerobic physical fitness performance. Future studies can use muscle quality or body fat classification as predictors of obesity in older adults, which could more precisely portray the relationship between obesity and health-related physical fitness performance.

## Data Availability

The data that support the findings of this study are available from [the Sports Cloud: Information and Application Research Center of Sports for All, Sport Administration, Ministry of Education in Taiwan] but restrictions apply to the availability of these data, which were used under license for the current study, and so are not publicly available. Data are however available from the authors upon reasonable request and with permission of [the Sports Cloud: Information and Application Research Center of Sports for All, Sport Administration, Ministry of Education in Taiwan].

## Appendix

**Table 7:** The percentiles of health-related physical fitness measurements in Taiwanese older adults

Variables	P_25_	P_33.33_	P_50_	P_66.66_	P_75_
**Men**					
2-min step test (step)	73	80	90	99	103
30-s arm curl test (rep)	14	15	18	20	22
30-s chair stand test (rep)	12	13	15	17	18
Back scratch test (cm)	-21	−17	−10	−4	0
Chair sit-and-reach test (cm)	−2	0	1	5	8
8-ft up-and-go test (s)	5.8	6.1	6.9	7.8	8.3
One-leg stance with eye open test (s)	5.0	7.0	12.8	24.7	30.0
**Women**					
2-min step test (step)	69	76	88	97	101
30-s arm curl test (rep)	14	15	17	20	21
30-s chair stand test (rep)	11	12	14	16	18
Back scratch test (cm)	−12	−8	−1	1	3
Chair sit-and-reach test (cm)	0	1	4	8	11
8-ft up-and-go test (s)	6.0	6.4	7.1	8.0	8.6
One-leg stance with eye open test (s)	4.4	6.0	10.8	20.0	27.0

Note: P_25_, P_33.33_, P_50_, P_66.66_, and P_75_ representing the location of the percentiles in data distribution

## Abbreviations

WC: Waist circumference
HC: Hip circumference
NTD: New Taiwan Dollar
BMI: Body mass index
WHR: Waist-to-hip ratio
SAS: Statistical Analysis System
ANOVA: Analysis of variance
OR: Odds ratio
CI: Confidence interval
